# Methylation State of the *EDA* Gene Promoter in Chinese X-Linked Hypohidrotic Ectodermal Dysplasia Carriers

**DOI:** 10.1371/journal.pone.0062203

**Published:** 2013-04-23

**Authors:** Wei Yin, Xiaoqian Ye, Huali Fan, Zhuan Bian

**Affiliations:** 1 The State Key Laboratory Breeding Base of Basic Science of Stomatology (Hubei-MOST) and Key Laboratory of Oral Biomedicine Ministry of Education, School and Hospital of Stomatology, Wuhan University, Wuhan, Hubei, China; 2 Department of Endodontics and Periodontics, College of Stomatology, Dalian Medical University, Dalian, Liaoning, China; 3 The Second General Department, Hangzhou Stomatology Hospital, Hangzhou, Zhejiang, China; Instituto de Ciencia de Materiales de Madrid - Instituto de Biomedicina de Valencia, Spain

## Abstract

**Introduction:**

Hypodontia, hypohidrosis, sparse hair and characteristic faces are the main characters of X-linked hypohidrotic ectodermal dysplasia (XLHED) which is caused by genetic ectodysplasin A (*EDA*) deficiency. Heterozygous female carriers tend to have mild to moderate XLHED phenotype, even though 30% of them present no obvious symptom.

**Methods:**

A large Chinese XLHED family was reported and the entire coding region and exon–intron boundaries of *EDA* gene were sequenced. To elucidate the mechanism for carriers’ tempered phenotype, we analyzed the methylation level on four sites of the promoter of *EDA* by the pyrosequencing system.

**Results:**

A known frameshift mutation (c.573–574 insT) was found in this pedigree. Combined with the pedigrees we reported before, 120 samples comprised of 23 carrier females from 11 families and 97 healthy females were analyzed for the methylation state of *EDA* promoter. Within 95% confidence interval (CI), 18 (78.26%) carriers were hypermethylated at these 4 sites.

**Conclusion:**

Chinese XLHED carriers often have a hypermethylated EDA promoter.

## Introduction

Mutations in *EDA* gene can lead to X-linked hypohidrotic ectodermal dysplasia (XLHED), the most common form of ectodermal dysplasias (EDs). The incidence is less than one in per 100’000 [1]. Mutant *EDA* affects cell signaling transduction or cell migration during the epithelial-mesenchymal inductive process. The structures of ectodermal origin are affected. Patients with XLHED have prominent clinical features: sparse hair, eyelashes and eyebrows, small, misshapen or missing teeth, diminished sweating with a history of high fevers in hot weather, decreased salivary secretions, and a characteristic special facial appearance. Facial features include prominent forehead, narrow and short maxillary regions, small palatal depth, small cranial length, and depressed nasal root and bridge of the nose. However most heterozygous carriers only show minor to moderate degrees of these abnormalities [2].

DNA methylation refers to the biological process that a methyl group added to cytosine that stands directly before a guanine molecule by DNA methyltransferases after DNA duplication. In living cells, methylation has been reported as one of the most common covalent modifications of DNA. It has both epigenetic and mutagenic effects on specific gene expression, cell differentiation, chromatin inactivation, embryo growth, and cancer.

*EDA* contains a large CpG island in its promoter. CpG islands located in gene promoters represent a major target for DNA hypermethylation, which impairs transcription upon regional or specific methylation events. It has been confirmed that promoter CpG island hypermethylation contributed to gene silencing by inhibiting the binding of certain transcription factors to their recognition sequence, attracting methylated DNA-binding proteins, and/or through chromatin remodeling [3]–[11]. However, whether aberrant methylation is related to XLHED carriers’ phenotype has been controversial.

In the present study, we report a causative *EDA* mutation (c. 573–574 insT) in a Chinese XLHED family. We investigated the methylation of *EDA* promoter of this family’ carrier as well as other 22 carriers we reported before [12], [13] to study correlations between the phenotype of carriers and the methylation state of the promoter. *EDA* gene in eighteen (78.26%) of the carriers were hypermethylated, and this result demonstrated that there was a correlation between being a XLHED carrier and the hypermethylation status of the *EDA* promoter.

## Materials and Methods

### Ethical Approval

This study was approved by the Institutional Review Board (IRB) of Hospital and School of Stomatology, Wuhan University. Written informed consents were obtained from all participants or their guardians.

### Nomenclature

Gene mutation nomenclature used in this study follows the recommendations of den Dunnen and Antonarakis Gene symbols used in this article follow the protocol created by the HUGO gene nomenclature committee [15].

### DNA Sample Collection

All probands were outpatient cases of School and Hospital of Stomatology, Wuhan University. Two professional dentists examined the patients respectively according to the classical diagnosis criteria. Comprehensive physical examinations and panoramic radiograph films were taken thoroughly. Pilocarpine iontophoresis sweat test was used to evaluate the function of sweat gland. – to ++ was scored for normal to absolutely no sweat. After a definite diagnosis was obtained, patients’ family members were interviewed and examined. 5–10 ml blood samples were collected with EDTA 2Na^+^ and heparin as anticoagulant. DNA was isolated from leukocytes using the standard sodium dodecyl sulphate–proteinase K–phenol/chloroform method After quality accessing, DNA was frozen at −20°C.

### Polymerase Chain Reaction (PCR) and Mutation Screening

The entire *EDA* coding region and exon–intron boundaries of patients and their relatives were amplified using the same primers used in the previous report Amplified fragments were purified with a PCR purification kit (Omega, USA) according to the manufacturer’s protocol. DNA sequences were obtained from both strands with an ABI PRISM 3730 genetic analyzer. Sequence analysis was performed by the CHROMAS program and BLAST program of the National Center for Biotechnology Information (NCBI). After identifying nucleotide variants in the *EDA* gene, 200 unrelated controls were examined respectively.

### Quantification of *EDA* Promoter’s Methylation State by Pyrosequencing

Bisulfite modification of the genome was processed with the CpGenome DNA Modification Kit (Intergen Company, Purchase, NY). The potential methylation sites analyzed in this study were “CGgctgaggcagaCGcagCGgctccCG” located in *EDA* promoter. Specific amplification and sequencing primers were designed by PSQ Assay Design 1.0. One of the PCR primers was labeled with biotin. Blank sample was used as negative control. 97 clinically healthy subjects who had similar ages as carriers were recruited as normal controls.

Pyrosequencing analysis was conducted with PYRO MARK ID (BIOTAGE). The figures and data were analyzed by PSQ96MA 2.1 software. Peak value in the sequencing picture means allele frequency of the DNA sample. The percentage of residual C (Cm) showed how much of each site has been methylated. To insure the reliability of the results, all of the samples underwent pyrosequencing twice.

### Statistical Analysis

Taking advantage of parameter estimation, 95% confidence interval (CI) was employed to do statistical analysis. Within CI, carriers were divided into 3 groups, hypermethylation, normal and hypomethylation groups.

## Results

### Clinical Data

**Figure 1:**
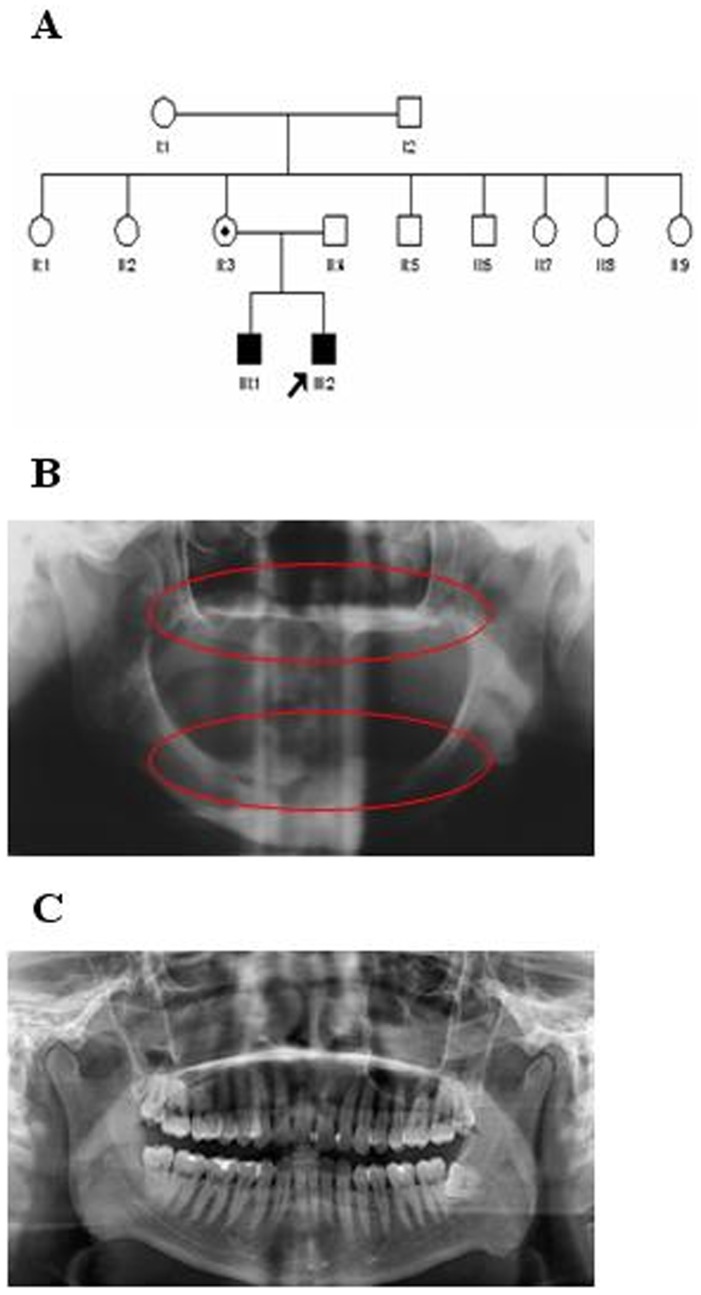
Pedigree and tooth development features of the Chinese family. (A) Males are indicated by squares, females by circles. Affected individuals are indicated by filled symbols and unaffected individuals by white symbols. Circle containing a dot refers to carrier. An arrow indicates the proband. (B) The panoramic radiographs of the proband confirmed there was no tooth germ in the alveolar bone (red circle) which was the severest symptom of tooth dysplasia. (C) The panoramic radiographs of a healthy control with normal tooth development.

The clinical features of this Chinese family are listed in Table 1. The 2 patients experienced the classic XLHED symptoms, hypodontia, sparse hair, thin and dry skin and specific facial features (Fig. 1). Both of them were born by a normal delivery, and had normal psychomotor and intellectual development.

**Table 1:** Clinical phenotypes of family members in the Chinese pedigree.

Family ID	Person ID	Gender	Affection status	Age (years)	Skin		Sparse hair	Hypohidrosis	Facial features	Eczema	Tooth existing (Hypodontia)	Nail dysplasia	Sweat glands dysplasia	Other Manifestations
					Hypoplastic	Thin & wrinkled								
I	II3	F	C	50	–	–	+	–	–	–	NM	–	–	
I	III1	M	A	25	++	++	++	+	+	+	13, 23	+	++	
I	III2	M	A	23	++	++	++	++	+	+	anodontia	+	++	

A: affected; C: carrier; F: female; M: male; NM: no missing; +: positive; number of ‘+’ symbols reflects the degree of these clinical features; -: negative; 13: Right maxillary canine; 23: Left maxillary canine.

The carrier (II3) had no clinical abnormity except for sparse hair. The other 22 carriers we reported before had a similar trait. The most common features were sparse hair and aberrant tooth shape. All of them were Han people which account for 90.56% of the population in China and lived in the middle of China, ranging from teens to 60s in age. They harbored 8 missense mutations, 1 frameshift mutation, 1 splicing site mutation and 2 exon deletions.

### Identification of the Causative *EDA* Mutation

**Figure 2:**
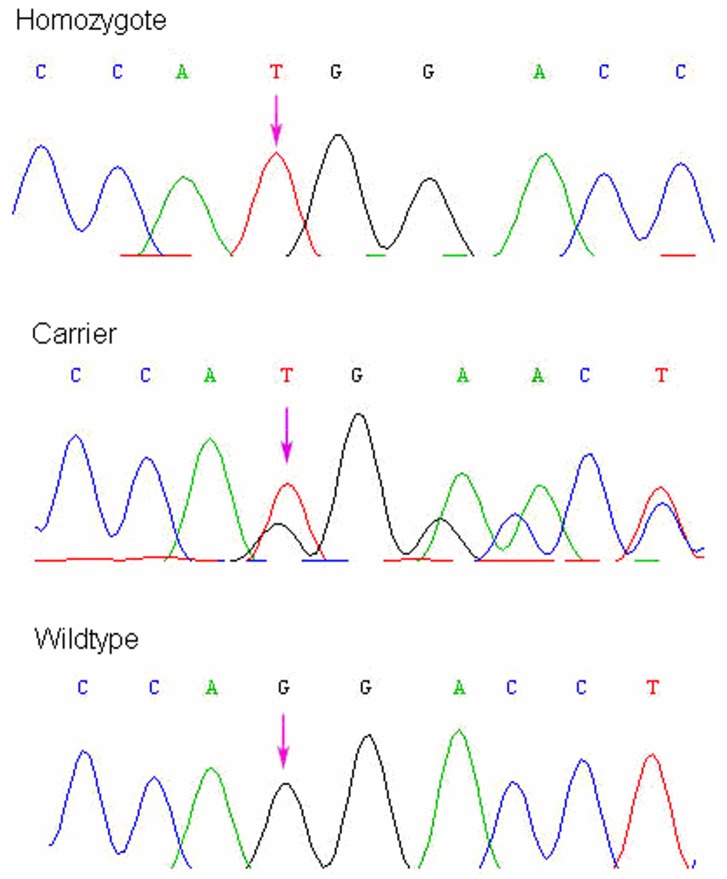
Identification of the causative mutation in *EDA* gene. Arrows indicate the mutation site. The affected male patient and his mother harbored a frameshift mutation c.573–574insT.

Direct sequencing was used to analyze all the coding exons and intron-exon boundaries from both directions. The proband had a nucleotide insertion in exon 5, c.573–574 insT (Fig. 2). His mother (II3) and elder brother (III1) also had the same mutation. The insertion induced a frameshift from amino acid 192 and caused the transcription to stop at amino acid 239. 200 healthy controls did not have this change.

### Features of *EDA* Promoter Methylation

**Figure 3:**
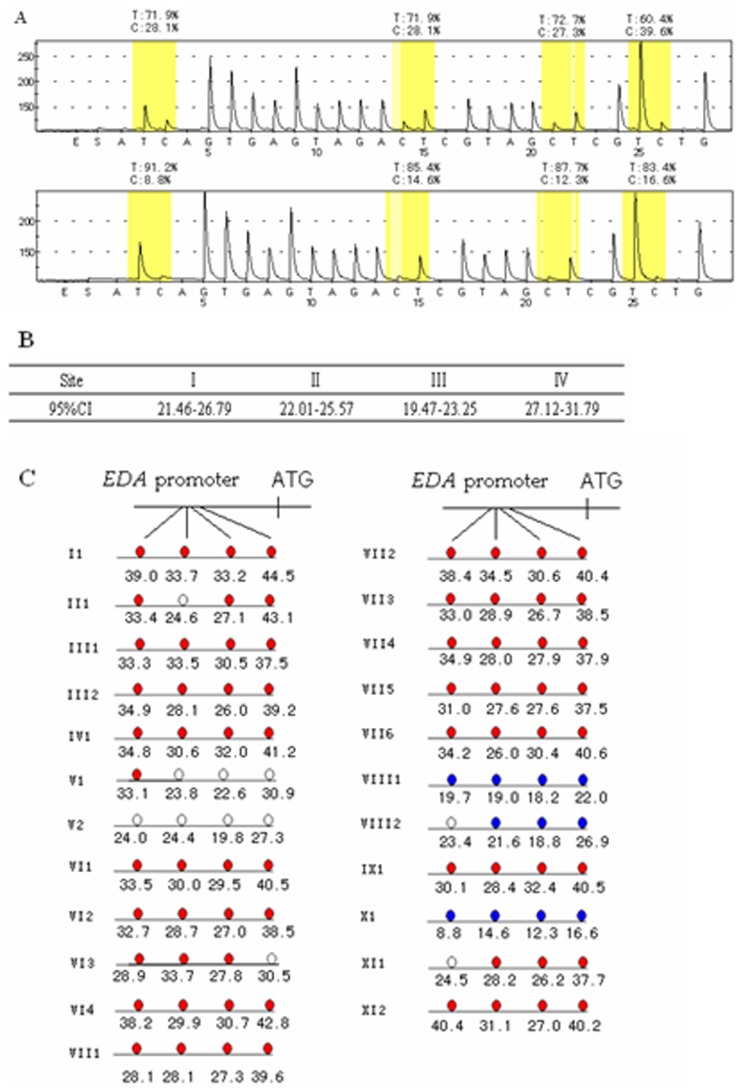
*EDA* promoter’s methylation analysis of 23 carriers. (A) Pyrosequencing graphs of 2 samples, a hypermethylation carrier and a hypomethylation carrier. Peak heights are proportional to the number of identical residues incorporated. Percentage in pictures means allele frequency of each site. (B) The 95% CI for the 4 sites. The figures which refer to methyl-cytosine percent at that site are calculated as described in the text. (C) The methylation state of each carriers in the 4 sites. Red, white and blue refer to hypermethylation, normal and hypomethylation respectively.

**Figure 4:**
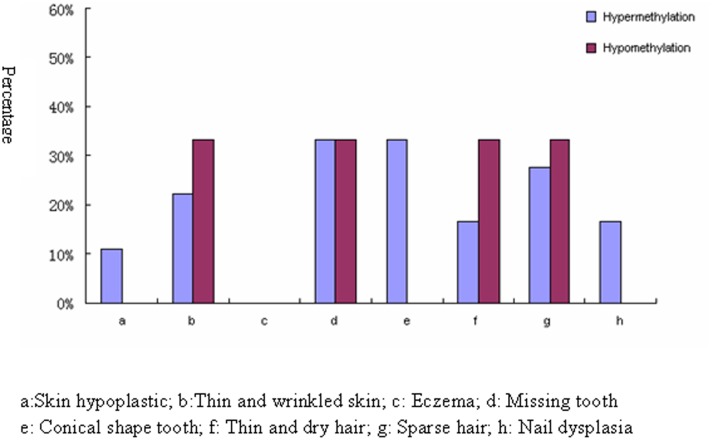
Relationship between methylated state and phenotype of XLHED carriers. Hypermethylated carriers are inclined to have more conical shaped tooth and nail dysplasia than hypomethylated group.

The 95% CI for methylation of each site is shown in Fig. 3B. Compared with it, 78.26% (n = 18) carriers were in hypermethylated state while 14.29% (n = 3) were hypomethylated. The other 2 samples were in normal state. The methylation state had no correlation with carriers’ mutation type and site (Table 2). Similarly, methylation state and phenotype also had no correlation. However, hypermethylated group was inclined to have more defects in nails and tooth shape when compared to hypomethylated carriers (Fig. 4).

**Table 2:** The mutations and some selected clinical findings of twenty-three carriers.

Familynumber	Exon/intron	Mutation type	Nucleotide changes	Amino acid change	Domain^a^	Sparse hair	Number of tooth missing	Conical shape tooth	Nail dysplasia	Sweat glands dysplasia	Methylation state^b^ (Cm value)
II1^[12]^	1	Missense	c.200A>T	E67V	E	–	1	0	–	–	Hyper
III1^[12]^	3	Missense	c.463C>T	R155C	F	+	5	0	+	NE^c^	Hyper
III2^[12]^	3	Missense	c.463C>T	R155C	F	–	NE^c^	NE^c^	–	–	Hyper
IV1^[13]^	3	Missense	c.467G>A	R156H	F	–	0	0	–	–	Hyper
V1^[12]^	3	Missense	c.491A>C	E164A	E	+	0	0	-	–	Normal
V2^[12]^	3	Missense	c.491A>C	E164A	E	+	0	0	+	–	Normal
VI1*	3	Deletion	–	–	E	+	7	0	+	NE^c^	Hyper
VI2*	3	Deletion	–	–	E	+	7	1	–	–	Hyper
VI3*	3	Deletion	–	–	E	–	0	0	–	–	Hyper
VI4*	3	Deletion	–	–	E	–	0	2	–	–	Hyper
VII1*	3	Deletion	–	–	E	–	0	0	–	–	Hyper
VII2*	3	Deletion	–	–	E	–	0	0	–	–	Hyper
VII3*	3	Deletion	–	–	E	–	0	4	–	–	Hyper
VII4*	3	Deletion	–	–	E	–	0	4	–	–	Hyper
VII5*	3	Deletion	–	–	E	–	0	2	–	–	Hyper
VII6*	3	Deletion	–	–	E	–	0	2	–	–	Hyper
I1	5	Frameshift	573insT	FS at 192 Term	C	–	0	0	–	–	Hyper
VIII1^[12]^	5	Splice donor site	IVS5+1 g>a	Altered splicing	C	–	0	0	–	–	Hypo
VIII2^[12]^	5	Splice donor site	IVS5+1 g>a	Altered splicing	C	–	0	0	–	–	Hypo
IX1^[12]^	7	Missense	c.758T>C	L253P	T	+	9	0	+	–	Hyper
X1^[12]^	9	Missense	c.926T>G	V309G	T	+	3	0	–	–	Hypo
XI1^[13]^	9	Missense	c.1045G>A	A349T	T	+	6	0	–	–	Hyper
XI2^[13]^	9	Missense	c.1045G>A	A349T	T	–	0	0	–	–	Hyper

a E: Extracellular domain; F: Furin domain; C: Collagen domain; T: TNF homology domain.

b Hyper: hypermethylation; Hypo: hypomethylation.

c not examined.

* unpublished data.

## Discussion

EDA is a trimeric type II membrane protein that co-localizes with cytoskeletal structures at the lateral and apical surfaces of cells The protein includes intracellular domain, transmembrane domain, furin subdomain, collagen subdomain and TNF homology subdomain As a member of the TNF-related ligand family, EDA is involved in the early epithelial-mesenchymal interaction So far hundreds of mutations have been identified. About 80% of them are small intragenic changes, including point mutations, small deletions and insertions, and more than half of them are found in exons 1, 3 and 5. Large deletions, including entire exon loss and complete gene deletion, have also been reported But the type of mutations, the phenotype and disease severity showed no obvious correlation especially for heterozygous carriers. About 30% of them do not even have obvious symptoms, rendering accurate diagnosis of carrier status difficult [2].

In this study, we reported a known frameshift mutation in the *EDA* gene. The frameshift mutation, c.573–574insT, caused aberrant transcription from codon192 and a premature stop at 239. So far, at least 14 frameshift mutations have been reported in exon 5, but only 3 were insertions. Mutant *EDA* missed partial collagen subdomain and the whole TNF homology subdomain. The TNF homology domain consisted of 10 predicted antiparallel b-sheets linked by variable loops, in common with other members of the TNF family, which was necessary for the homotrimerization of ligands and the binding of *EDA* to its receptor Therefore the mutant *EDA* was predicted not to bind *EDAR* at all.

In the post-genomic era, it is becoming increasingly evident that epigenetic controlling of gene expression plays an important role in determining the phenotype. Histone modifications and DNA methylation-demethylation events are central to the epigenetic regulations of development XLHED female carriers are mosaics of functionally normal and abnormal cells. The carriers’ clinical features are likely to depend on the percentage of abnormal cells having participated to the process of ectodermal appendage formation. But it is presently unclear whether EDA promoter methylation contributes at all to the phenotype of carriers.

To obtain more precise hints, we chose the quantitative method, pyrosequencing system [22], [23], to analyze the methylation level of *EDA* promoter. Besides the carrier of this family, we additionally recruited 22 other carriers that we reported before. Most of them showed mild symptoms of tooth and hair impairments. All of the causative mutations were distributed in the extracellular domain. 18 of the carriers displayed hypermethylation of the *EDA* promoter. Some of them were even 50% higher in methylation level than normal controls. Although hypermethylated carriers were inclined to have more conical shaped tooth and nail dysplasia than that of hypomethylated group, no regular pattern seemed to exist among methylation state, mutation type, mutation site and clinical features. Some hypermethylated carriers appeared clinically normal, but some even had as many as 7 missing teeth, conical tooth and sparse hair. The 3 hypomethylated carriers came from 2 families. The 2 carriers who had a mutation in splice donor site were totally normal. The other carrier had sparse hair and missing tooth. Her missense mutation was located at the end of the transcript. The phenotypic changes may be due to the modulation of selection at another X chromosome locus or polymorphism at a locus controlling inactivation. The 2 methylation normal individuals came from the same family. They exhibited mild sparse hair. The mutation occurred between the furin cleavage site and the collagen-like domain.

In addition, the measure of promotor’s methylation by pyrosequencing is for both WT and mutated allele on both active and inactive X chromosomes, further studies are needed to explain whether this effect is specific to the *EDA* promoter or is generalized to the entire X chromosome.

In conclusion, a recurrent *EDA* missense mutation, c.573–574 insT in a Chinese patient was reported. We also performed the first study to elucidate the variation of DNA methylation patterns in *EDA* promoters on Chinese XLHED carriers. The results suggest most of these Chinese XLHED carriers’ have hypermethylated EDA promoter.
