# Correction: Methylation State of the *EDA* Gene Promoter in Chinese X-Linked Hypohidrotic Ectodermal Dysplasia Carriers

**DOI:** 10.1371/annotation/4d6c4127-82e4-408d-af89-5f2e207d523b

**Published:** 2013-09-09

**Authors:** Wei Yin, Xiaoqian Ye, Huali Fan, Zhuan Bian

A funding organization and grant were incorrectly omitted from the Funding Statement. The Funding Statement should read: "This work was supported by grants from the National Natural Scientific Foundation of China (30500562, 30772417, 30930099 (Monumental Projects) and 81120108010 (International cooperation and exchange projects)), the Pre-National Basic Research Program of China (973 Plan) (2012CB722404), the Chenguang Plan for Distinguished Youth of Wuhan, China (200850731374), the Open Research Fund Program of Hubei-MOST KLOS & KLOBME (201103), the Foundation for the Author of National Excellent Doctoral Dissertation (2007B69) and the Program for New Century Excellent Talents in University, Ministry of Education, China. The funders had no role in study design, data collection and analysis, decision to publish, or preparation of the manuscript." 

